# Bayesian-based decipherment of in-depth information in bacterial chemical sensing beyond pleasant/unpleasant responses

**DOI:** 10.1038/s41598-022-06732-4

**Published:** 2022-02-22

**Authors:** Hiroto Tanaka, Yasuaki Kazuta, Yasushi Naruse, Yukihiro Tominari, Hiroaki Umehara, Yoshiyuki Sowa, Takashi Sagawa, Kazuhiro Oiwa, Masato Okada, Ikuro Kawagishi, Hiroaki Kojima

**Affiliations:** 1 Advanced ICT Research Institute, National Institute of Information and Communications Technology, Kobe, Hyogo 651-2492 Japan; 2 Center for Information and Neural Networks (CiNet), National Institute of Information and Communications Technology and Osaka University, Kobe, Hyogo 651-2492 Japan; 3 Department of Frontier Bioscience and Research Center for Micro-Nano Technology, Hosei University, Tokyo, 184-8584 Japan; 4 Graduate School of Life Science, University of Hyogo, Harima Science Park City, Hyogo 678-1297 Japan; 5 Graduate School of Frontier Sciences, The University of Tokyo, Kashiwa, 277-8561 Japan

**Keywords:** Biomimetics, Sensors and probes, Nanobiotechnology, Biosensors, Biological techniques, Biotechnology, Microbiology, Bacteria, Cellular microbiology, Nanobiotechnology, Biosensors, Biophysics

## Abstract

Chemical sensing is vital to the survival of all organisms. Bacterial chemotaxis is conducted by multiple receptors that sense chemicals to regulate a single signalling system controlling the transition between the direction (clockwise vs. counterclockwise) of flagellar rotation. Such an integrated system seems better suited to judge chemicals as either favourable or unfavourable, but not for identification purposes though differences in their affinities to the receptors may cause difference in response strength. Here, an experimental setup was developed to monitor behaviours of multiple cells stimulated simultaneously as well as a statistical framework based on Bayesian inferences. Although responses of individual cells varied substantially, ensemble averaging of the time courses seemed characteristic to attractant species, indicating we can extract information of input chemical species from responses of the bacterium. Furthermore, two similar, but distinct, beverages elicited attractant responses of cells with profiles distinguishable with the Bayesian procedure. These results provide a basis for novel bio-inspired sensors that could be used with other cell types to sense wider ranges of chemicals.

## Introduction

In higher organisms, a sophisticated neural network is essential for the identification of chemical substances. In mammalian olfaction, for instance, tens of thousands chemicals can be identified by only hundreds of receptors, each of which is expressed in a set of olfactory cells with axons projecting to the same glomeruli^1^. However, it has not been addressed experimentally whether a neural network or an array of sensory cells with distinct receptors is essential for the detection of individual chemicals.

**Figure 1:**
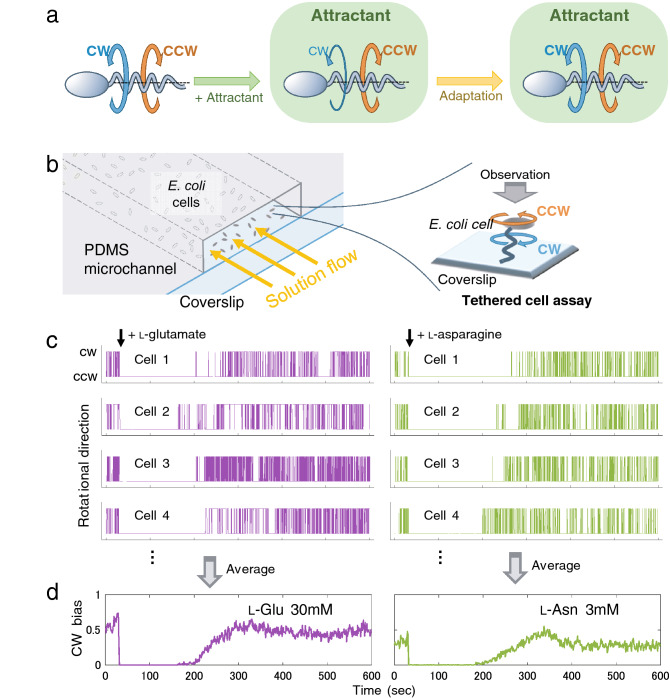
Experimental design. (**a**) Each flagellar motor of *E. coli* cells in steady state environment shows bi-directional motions stochastically (CW or CCW direction). When an attractant is added to the environment (chemical stimulus), cells detect differences in the amount of the attractant and bias flagellar rotation in the CCW direction. After a sufficient time (~ 400 s in case of **c**, **d**), under constant concentrations of attractants, each flagellar motor recovers bi-directional motion (adaptation). (**b**) Schematic drawing of the experimental setup (left) and tethered cell assay (right). Each experimental chamber consisted of a PDMS microchannel on a coverslip. *E. coli* cells applied to a chamber were adhered to a glass surface via cell bodies and/or flagella. The rotational motion of cell bodies, each tethered via flagellum by chance, was analysed. (**c**) Rotational directions of individual cells. Cells were stimulated by (left column) l-Glu (30 mM) and (right) l-Asn (3 mM). Vertical axes show the rotational directions (CW or CCW). Horizontal axes are common to (**d**) and represent time. Arrows indicate the times of chemical stimulations. We measured rotational motions of cells at every 10 ms (1 frame of our movies), and counted those more than 7.5 degrees/frame, which was more than two times larger than that of noise (< 3 degrees/frame). Most of cells rotated CCW immediately after chemical stimulation (attractant response) and recovered bi-directional rotations as in the initial phase over time (adaptation). Individual cells had varied response time courses. (**d**) Ensemble averages of rotational directions of cells. Vertical axes represent the CW bias, which was calculated as the fraction of CW motion (number of CW rotations) to all rotational motions (number of CW + CCW motions) at 1-s intervals. The motions of more than 100 of approximately 500 observed cells were averaged.

*Escherichia coli*, a unicellular organism, detects chemicals, processes chemical information and preforms various activities in response. *Escherichia coli* cells have machineries to respond to environmental chemicals^2–5^. Here, we focus on the chemotactic behaviour of *E. coli* to test what sort of information we can extract from responses of *E. coli* cells. Chemotaxis of *E. coli*, which is a swimming movement toward attractants or away from repellents via flagellar rotation, has been extensively studied for decades^5–8^ (Fig. 1a). All components involved in this process have been identified and characterised. Transmembrane sensor proteins, i.e. Tsr for serine and repellents, Tar for aspartate, maltose and repellents, Trg for ribose and galactose, Tap for dipeptides and Aer for redox state, detect various stimuli either directly or with the help of soluble receptors^9^. An intracellular signal pathway integrates input signals to modulate the phosphorylation level of the response regulator protein CheY, which controls switching of the rotational direction of the flagellar motor. Attractant stimuli increase the probability of counterclockwise (CCW) rotation, whereas repellents augment clockwise (CW) rotation (Fig. S1a).

Single cell responses to various stimuli can be examined, for instance, with the use of a tethered cell assay^10^, in which the rotation of a single flagellar motor is monitored as the rotation of the cell body (Fig. 1b). However, the responses of individual *E. coli* cells, even if genetically uniform, highly differ. The individuality of bacterial cells has long been recognised^11^ and is presumably due to non-genetic variations of the levels of various proteins and metabolites. Indeed, recent comprehensive studies have revealed a wide range of cell-to-cell variation in the expression level of any gene^12–16^. Despite such individuality, *E. coli* cells, as a population, perform reproducible chemotactic responses that seem idiosyncratic to attractant chemical species. Here, to reveal basic design of the chemotaxis system of *E. coli*, the collective responses were examined in detail by ensemble averaging of the behaviours of many individual cells to similar, but distinct, attractants.

## Results

### Experimental design

To analyse the collective responses of *E. coli* cells with reasonable accuracy, an experimental setup was constructed to monitor the responses of multiple cells stimulated simultaneously with the use of a tethered cell assay^10^, in which the rotation of a single flagellar motor is monitored as the rotation of the cell body (Fig. 1b). Under optimised conditions, more than 100 of approximately 500 of *E. coli* cells observed in each microscopic field were found to be rotating. Rotating cells are considered to be fixed on the surface through only one flagellum, and we extracted this population and analysed. The rotational directions of the cells were analysed concurrently at 10-ms intervals with custom-made image and motion analysis programs (Fig. S2). Care was taken to reduce the influence of the viscous drag force of the solution by minimising the flow rate (see the supplementary information [SI] for details, Fig. S3). Using this setup, the responses of many cells to two standard amino acid attractants, l-glutamate (l-Glu) and l-asparagine (l-Asn), were measured. When exposed to either attractant, each cell exclusively rotated CCW and, within a relatively short time, resumed pre-stimulation behaviours, i.e. alternating the rotational direction between CCW and CW (Fig. 1c). However, the responses of individual cells to even the same attractant substantially differed in terms of the time course of stimulation and adaptation. Nevertheless, ensemble averaging of a large set of ‘noisy’ data revealed essentially similar, but apparently distinct, traces of the two attractants (Fig. 1d).

### Comparison of ensemble averaged responses of *E. coli* cells to different attractants

**Figure 2:**
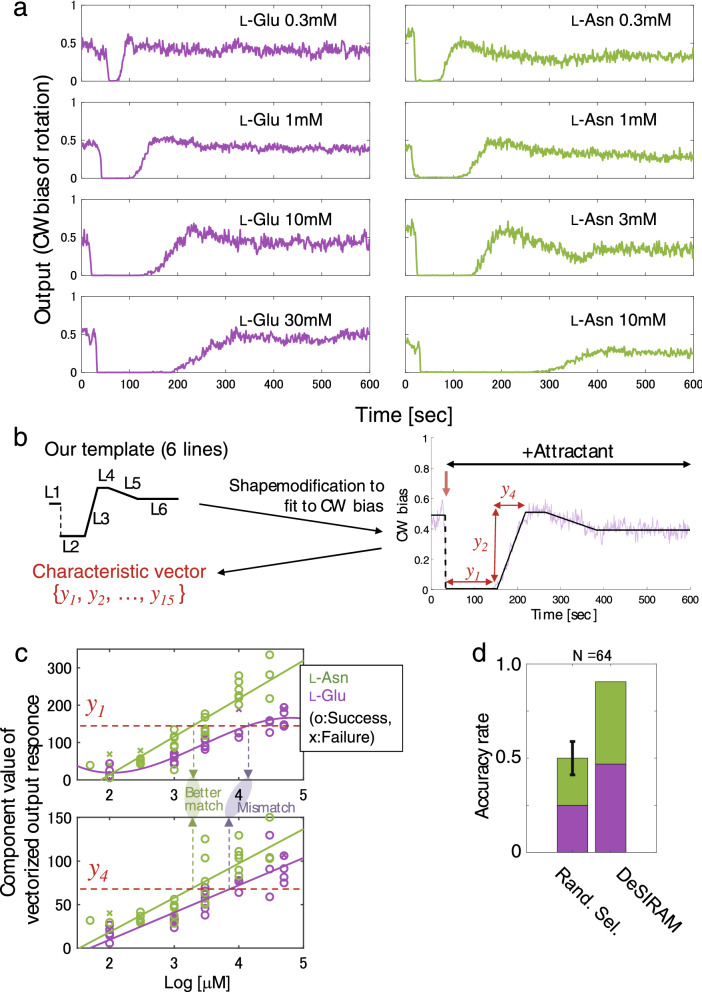
Decipherment of two input chemicals from output responses of *E. coli* cells. (**a**) Typical responses to l-Glu (left column) and l-Asn (right column). Output traces of CW bias show a common profile consisting of a strong attractant response immediately after chemical stimulation (CW bias ≅ 0.0) and a recovery phase returning to near initial CW bias after sufficient time. Each graph is coloured according to chemical species (purple, l-Glu; green, l-Asn). The concentrations of chemicals increase from top to bottom. (**b**) Construction of characteristic vectors. Individual vectors representing each CW bias trace were calculated by matching a geometric template consisting of 6 lines (L1–L6). The template shape (both amplitudes and durations of lines) was modified to fit CW bias traces and 15 arbitrary parameters were obtained (see SI for details). As examples, parameters of y_1_, y_2_ and y_4_ are shown, where y1 is the duration of L2 and y_2_ and y_4_ are the amplitude and duration of L3, respectively. (**c**) Concentration dependencies of 2 indexes, $y_{1} , y_{4}$, of characteristic vectors (all in Fig. S5). The values of $y_{1} ,\; y_{4}$ (positions are indicated in **b**) of characteristic vectors obtained as response activities to l-Glu (purple) and l-Asn (green) are plotted. Each graph is coloured according to chemical species as in (**a**). Plot makers of ‘o’ indicate data used to successfully identify the blind samples, whereas ‘x’ indicates failure. Solid lines show model functions representing concentration dependencies. Based on these model functions, Bayesian estimation is performed using observation values of blind samples, and we decipher type of blind sample (see text for details). Dashed red lines, green and purple arrows are added as examples of observations for explanation. The accuracy rates of decipherment and model functions were evaluated with leave-one-out cross validation. (**d**) Accuracy rate of decipherment for attractants groups consisting of l-Glu (n = 32) and l-Asn (n = 32). The left bar shows the theoretical accuracy rate of RS (≅ 1/2). The black line on left bar represents the standard deviation calculated numerically (± 0.09 =  ± 5.7/64, see Table S1 and SI for details). The right bar shows the accuracy rate by DeSIRAM (decipherment accuracy = 0.91). The probability of an accurate identification rate of 0.91 by RS was 4.1 × 10^−12^. This small probability confirms the validity of the procedure.

Further analyses of the collective responses of *E. coli* cells to different attractants were conducted to determine whether the attractant responses were chemical-specific. First, the attractant responses (time courses of mean CW bias values) of more than 100 cells (hereinafter referred to as CW bias traces) to various concentrations of l-Glu and l-Asn were measured (Fig. 2a). Although the strength of stimulation differed between the two attractants, the higher the amino acid concentration, the longer the attractant response persisted (CW bias ≅ 0) (Fig. 2a). Next, for mathematical treatments, the intracellular signalling system was treated as a black box and each CW bias trace as a geometric figure that is presumably described by a simplistic template. Each time course of CW bias was fitted by a combination of six linear lines (Figs. 2b and S4, L1–L6), where L1 represents the pre-stimulation state, L2 the stimulated state, L3 to L5 the recovery processes and L6 the post-adaptation state. Consequently, the trace was converted to a vector consisting of 15 index values $\left\{ {y_{1} , \ldots , y_{15} } \right\}$ (see SI for details) to collect training data of responses to each chemical, where y1 is the duration of L2 and y2 and y3 are the amplitude and slope of L3, respectively. In spite of the large deviations among cells that presumably result from preparation-to-preparation variations, the distributions of these index values differed between the attractant species, which are presented as different coloured data points in Fig. 2c. These results raised the possibility that information could be extracted to identity the input stimulation (chemical species and concentration) by analysing the ensemble averaged time course of CW bias in one way or another.

### Use of a Bayesian inference framework to analyse ensemble averaged responses

To deduce the identity of input stimulation out of complicated output responses (i.e. excitation-adaptation time courses), a statistical framework, **De**cipherment procedure of chemical **S**timulus **I**nversely from **R**esponse **A**ctivities with **M**achine learning (DeSIRAM), was developed based on Bayesian inference and supported by machine leaning. The index values indeed showed characteristic dependencies on the attractant species and concentrations (Fig. 2c). In DeSIRAM, we acquire model functions from the training data set prepared with various concentrations of sample chemicals (Figs. 2c, 4b and SI). Using these functions as templates for Bayesian inference, we can perform discrimination of species from blind sample with unknown concentration and species. Here is a brief explanation of our chemical type deciphering procedure. Consider a case where parameter values of red dashed lines (y_1_ = 150, y_4_ = 70) are observed as a result of stimulation with a blind sample. Comparing sample concentrations (green and purple arrows in upper and lower panels in Fig. 2c) estimated from the two models of l-Asn case and l-Glu case (green and purple lines), l-Asn shows a closer concentration value as the estimated value. In this case, the blind sample is likely to be l-Asn. These probabilities are calculated by Eq. (1). Figure 2d shows the rates of accurate identification of chemical species (l-Glu or l-Asn) with the use of DeSIRAM or random selection (RS) (see SI for details, the error bar indicates the theoretical standard deviation). The accurate identification rate obtained with DeSIRAM was significantly higher than by RS (0.91 vs. 0.50, respectively). The probability of an accurate identification rate of 0.91 by RS was 4.1 × 10^−12^. Such an extremely low probability clearly demonstrates that DeSIRAM can distinguish responses to the two attractants.

### Decipherment of input signals from collective responses of *E. coli* to well-defined attractants

**Figure 3:**
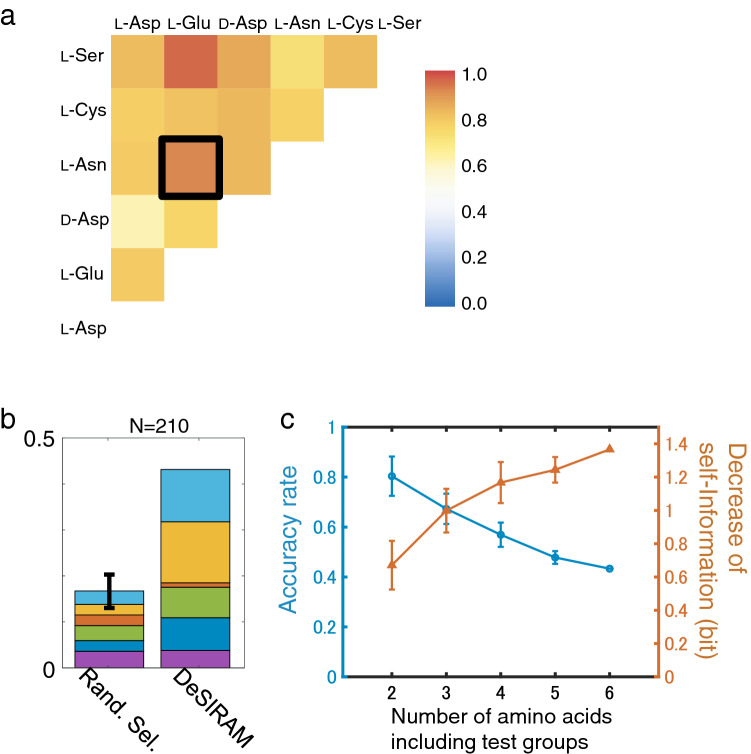
Discrimination of various attractants with DeSIRAM. (**a**) Accuracy rate spectrum of decipherment of chemical types of blind samples using DeSIRAM. Accuracy rates of decipherment of 15 groups consisting of 2 of 6 standard attractants. Vertical and horizontal axes indicate attractants included in the tested group. Six amino acid attractants are numbered arbitrary, l-Asp (1), l-Glu (2), d-Asp (3), l-Asn (4), l-Cys (5) and l-Ser (6). Black square shows data of Fig. 2d. (**b**) Accuracy rate of decipherment for attractant groups consisting of 6 standard attractants: l-Asp (dark blue), l-Glu (purple), d-Asp (red), l-Asn (green), l-Cys (yellow) and l-Ser (light blue). The left bar shows theoretical accuracy rate of RS (≅ 1/6 = 0.17). The black line on the left bar shows the standard deviation calculated numerically (= 0.04, see SI for details). The right bar shows accuracy rate by DeSIRAM. The decipherment accuracy of 0.43 is high relative to that by RS. The probability of an identification success rate of 0.43 by RS is 1.3 × 10^−19^. (**c**) Dependencies of accuracy rate (blue makers) and DSI (red markers) on number of attractant (NA) including in the tested groups. Plotted values are presented as averages and error bars indicated the standard deviations (n = 15, 20, 15, 6, 1 for NA = 2, 3, 4, 5, 6, respectively). Numbers of data (N) varies with NA. Here, 6 amino acids were prepared as standard attractants. For instance, in groups consisting of three amino acid attractants, N becomes _6_C_3_ = 20. The DSI of each group was calculated as, $DSI = - \left[ { - log_{2} \left( {accuracy\;rate\;by\;DeSIRAM} \right) - \left\{ { - log_{2} \left( {accuracy\;rate\;by\;RS} \right)} \right\}} \right]$, and, averaged in each NA group.

The performance of DeSIRAM was evaluated using six well-defined amino acid attractants with subtle differences in structure: i.e. l-aspartate (l-Asp), d-aspartate (d-Asp), l-Asn, l-Glu, l-cysteine (l-Cys) and l-serine (l-Ser) (Figs. S6 and S7). Here, 2 of 6 standard attractants were chosen and 15 (= _6_C_2_) groups were created. In all combinations of blind tests, DeSIRAM was able to deduce the identity of the input chemical with varied accuracy rates (such identification is hereinafter referred to as decipherment). Accuracy rates of decipherment by DeSIRAM were higher than those by RS (≅ 0.5) in all combinations (Figs. 3a and S8a, Table S1). Notably, even subtle changes in physicochemical properties which cannot be easily detected by artificial analysis, such as chirality (l-Asp vs. d-Asp) and the presence or absence of a single methylene group (l-Asp vs. l-Glu), were well discriminated. A blind test was also conducted with all six attractants at the same time. In this case, the accuracy rate of decipherment by DeSIRAM was 0.43, while the probability of accurate identification by RS was 1.3 × 10^−19^ (Fig. 3b, Table S5). The occurrence probability of the six-attractant group, which was much lower than that of any two-attractant group (Table S1), shows that DeSIRAM performed better in the former although the accuracy rate was lower. To compare groups consisting of 2, 3, 4, 5 and 6 standard attractants, values of self-information (i.e. entropy of a random variable) were calculated for all groups, where a decrease in self-information (DSI) corresponded to an increase in accuracy of decipherment performance. Although the accuracy rate decreased as the number of choices increased (Fig. 3c, blue makers), DSI increased as the number of attractants in a group increased (Fig. 3c, red makers). Thus, DeSIRAM works well to decipher information of input chemicals by analysing the chemotactic behaviour of *E. coli* cells, despite lacking complex detector arrays and neural networks.

The accuracy rates of DeSIRAM varied from 0.61 (discrimination of l-Asp and d-Asp) to 0.96 (l-Glu and l-Ser). This variation could be explained by types of relevant chemoreceptors. l-Glu and l-Ser are sensed mainly by Tar and Tsr receptors, respectively, whereas both l-Asp and d-Asp bind to Tar. The involvement of distinct receptors may cause distinct features in the response time course, which can be readily discriminated with relatively high accuracy. Notably, despite mainly activating the same receptors (Tar), the high rate of detection accuracy of l-Glu and l-Asn (black square in Fig. 3a) is remarkable and may indicate subtle differences in signal processing as well as the metabolism of individual chemicals.

### Decipherment of unorthodox chemical mixtures

**Figure 4:**
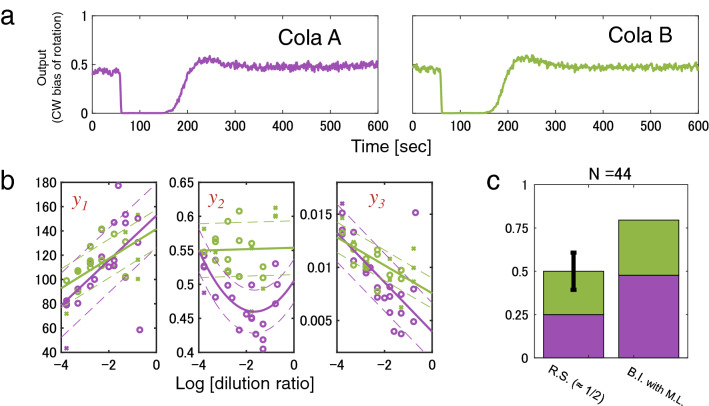
Discrimination of unidentified chemical mixtures with DeSIRAM. (**a**) CW bias traces caused by chemical composition stimuli of two similar, but distinct, cola beverages, cola A (left) and cola B (right). Chemotactic responses to solutions diluted to 1:2000. (**b**) Concentration dependencies of first 3 indexes, $y_{1} ,\;y_{2} ,\; y_{3}$, of characteristic vectors. Each graph is coloured according to chemical solution species (purple, cola A; green, cola B). Plot makers and lines have the same meaning as in Fig. 2c. (**c**) Accuracy rate of decipherment for chemical compositions (cola A, n = 22; cola B, n = 22). The left bar shows theoretical decipherment accuracy of RS. The black line on the left bar indicates the standard deviation calculated numerically. The right bar shows the accuracy rate by DeSIRAM. Decipherment accuracy of 0.80 is high relative to that of RS. The probability of an identification success rate of 0.80 by RS is 4.0 × 10^−5^. DeSIRAM with *E. coli* distinguished two solutions, although the compositions are not reported.

To test the versatility of DeSIRAM for decipherment of input chemicals, a blind test was performed between two similar, but distinct, cola beverages (cola A and B) that were likely never encountered before by the laboratory strain of *E. coli*. Interestingly, the cells responded sufficiently to both cola A and B even when diluted to 1:2000 (Fig. 4a). These traces were very similar and indistinguishable from one another. However, there was a significant difference in the concentration dependency of the index values of colas A and B (Fig. 4b). Accordingly, the accuracy of DeSIRAM to distinguish cola A from B was 0.80 (Fig. 4c). These results reinforce the notion that we can extract information of input chemicals from responses of *E. coli* cells to those chemicals. DeSIRAM can, therefore, also be applied to identify unusual chemical mixtures by analysing the response activities of organisms, rather than preparing an array of multiple cell types with distinct receptors.

## Discussion

The results of the present study revealed the chemical-specific responses of *E. coli* cells beyond simple strong/weak attractant/repellent categorisation of input stimuli. Although responses of individual cells varied substantially, the ensemble averaged response reflects identity of input chemical species. Namely, we can extract in-depth information from relatively noisy responses of *E. coli* cells to discriminate encountered chemical species and even their concentrations. As mentioned earlier, multiple sensors elicit signals to regulate the activity of the histidine kinase CheA and hence the phosphorylation level of the response regulator CheY, which modulates the CCW/CW rotation of the flagellar motor. Various chemicals are sensed by different receptors with diverse abundances, kinase-regulating abilities and methyl-accepting properties involved in adaptation. Alternatively, different chemicals can bind to a common receptor with dissimilar affinities. In either case, individual attractants have different effects on the time courses of the cellular level of phospho-CheY and hence CW bias of the flagellar motor. Although bacterial consumption of the input chemicals was negligible in the experimental setting, the effects of metabolism, permeability and toxicity of the chemicals on bacterial physiology should still be considered. Metabolism of input chemicals, for instance, may affect the intracellular concentration of acetylphosphate, which can serve as a direct phosphate donor to response regulators, including CheY^17–19^. The interplay of complicated signal transduction and metabolism networks produces chemical-specific responses. Furthermore, it has been reported that certain compounds (e.g. indole) affect swimming behaviour by directly binding to the flagellar motor^20^. Such compounds, if their abundances are modulated differentially during our observations, could also contribute the input chemical-specific responses. Moreover, this may lead to the application of our system to build a sensor based directly on motor output.

Whatever the molecular mechanisms, DeSIRAM, an analysis framework based on Bayesian inferences and machine learning, was able to identify input chemicals by detailed analyses of ensemble averaged output responses in spite of the wide range of deviations in responses of individual cells. DeSIRAM can be regarded as a conceptual prototype of cell-based sensors. For the development of such sensors, bacterial chemotaxis is suitable because of its instantaneous responses and binary output, as well as easy handling of bacterial cells. The proposed system can readily monitor the responses of many cells simultaneously with high fidelity and a short lag time. The system proved sufficiently sensitive for detection of differences in the concentrations and structures of the input chemicals. Moreover, it was able to apply to discrimination between mixtures of unknown chemicals as well.

A remarkable feature of the biosensor system is that the front end is equipped with living cells, rather than proteins or other biomaterials. Moreover, the evaluation axis of the input chemicals can be set arbitrarily as demonstrated by the discrimination of two cola beverages. A single *E. coli* strain with a wild-type chemotactic phenotype was used in all of the experiments. Use of various mutant strains, including those with altered metabolism or expressing engineered receptors for target chemicals, would help to optimise discrimination rates. Parallel processing of various types of cells in combination with the introduction of additional index values, such as those corresponding to speed and fluctuations in cellular rotation, could aid in the development of a novel system for appraisal of chemical information viewed from biological activities. Such improvements would meet application demands^21^, such as precise discrimination of chiral isomers and high throughput evaluation of complex environmental samples.

In conclusion, decipherment of input chemicals that drive bacterial chemotaxis from output responses can be achieved, with reasonable accuracy, by ensemble averaging of highly varied chemotactic responses of individual cells and application of a statistical framework based on Bayesian inferences and machine learning. These findings provide a basis for the development of bio-inspired sensors with the use of other cell types for the discrimination of wider ranges of chemicals^22–24^ to obtain information that is difficult to detect and quantify with conventional technologies.

## Material and methods

### Bacteria and culture conditions

*E. coli* strain SYC12 was derived from strain RP437, which has the wild-type chemotactic phenotype^25^ and carries the *fliC-sticky* allele. Cells were grown overnight from frozen aliquots (0.1 mL) in lysogeny broth and stored at − 80 °C in 5 mL of T-broth (1% Bacto tryptone, Difco Laboratories) with 10% (vol/vol) dimethyl sulfoxide and 0.5% sodium chloride at 30 °C for 5 h^26^.

### Polydimethylsiloxane (PDMS) experimental chamber

Custom-made flow chambers were used for observation of tethered cells. The chambers were made of PDMS micro channels bound to glass coverslips. The PDMS micro channel device was used to measure rotational motion over time by reducing the solution exchange flow speed^27^ in order to decrease the effects of physical perturbations that prevent rotation of the cell body, which allowed measurement of CW bias immediately after solution exchange (chemical stimuli) as shown in Figs. 1d and S2.

### Microscopy and imaging

Populations of *E. coli* cells were imaged using an inverted microscope Ti-2000 equipped with a Perfect Focus System (Nikon Corporation). A 20 × objective lens (Nikon CFI S Plan Fluor ELWD ADM20 × NA0.45) was used to obtain phase-contrast images of the cells. The large area image (approximately 400 µm × 300 µm) was recorded using a high-speed camera at 100 fps (LRH20000C-2741, Digimo Co., Ltd.).

### Tethered cell assay and image analysis

Cells were adhered to glass coverslips via sticky flagella filaments. Experiments were performed in motility buffer (10 mM potassium phosphate, pH 7.0, 0.1 mM ethylenediaminetetraacetic acid, 70 mM sodium chloride) containing one attractant amino acid at various concentrations (l-aspartic acid, 0.001–3 mM; l-Glu, 0.01–50 mM; d-aspartic acid, 0.01–50 mM; l-Asn, 0.05–30 mM; l-Cys, 0.01–3 mM; and l-Ser, 0.001–0.05 mM). All amino acid reagents were commercially available (Sigma-Aldrich). Buffer exchange was achieved by flowing 10 μL of motility buffer with or without amino acids via differences in hydrostatic pressure by varying the altitude of the reservoir. For acquisition data, motility buffer containing one of six standard attractant chemicals (training solution) was applied to the experimental chamber and output responses to the attractant stimuli were observed. All experiments were conducted at room temperature. For the conventional chemotaxis assay, the motility solution contained l-methionine and lactate, which allows for long-term (~ 6 h) observation^28,29^. However, in order to exclude any effect of these chemicals during the assay, no chemical was added to the motility solution. Therefore, the observation time was limited to 600 s.

Phase-contrast images of rotating cells were analysed by custom made program. The rotational direction of each cell was calculated based on the cell angles in sequential images. The CW bias of the cell population was calculated by averaging the rotational directions of all cells, which revealed an angular motion of > 7.5° after 1 frame (10 ms).

### Formulation of statistical procedure (DeSIRAM construction)

For decipherment of chemical stimuli, both the type and concentration of a blind sample are unknown. The chemotactic response is a multivariable function dependent on the type and concentration of chemicals, thus it is difficult to differentiate chemicals when lacking this information. In order to overcome this difficulty, a mathematical framework based on Bayesian inference^30^ was developed to classify the response vectors of a blind sample as a standard chemical substance.

Here, a briefly outline of the DeSIRAM framework is presented (see the SI for details). When $n$ types of chemical substances (chemical stimuli denoted by $s = 1,{ }2,{ } \ldots , N$) are prepared as a standard, the biological responses (example in Fig. 2a) of organisms to chemical $s$ are evaluated at a concentration of $x$. Here, CW bias trace was used as response activity and converted to 15 arbitrary characteristic values (a characteristic vector). As shown by the example in Fig. 2a, the CW bias trace is described with 6 lines (L1–L6). Generally, each chemical attractant (stimuli) provides one output vector, which is assumed to be characterised with a set of $m$ index values, $\left\{ {y_{i} } \right\}$, ($i = 1,{ }2,{ } \ldots , m$; in the present case, $m = 15$). Then, the chemical species, based on Bayesian inference, is deduced from observed $\left\{ {y_{i} } \right\}_{BTS}$ of a blinded test sample. Although $\left\{ {y_{i} } \right\}$ is affected by both the chemical species ($s$) and ${\text{concentration }}\left( x \right)$, the focus of this report was the determination of $s_{BTS}$. Therefore, the output probability distribution function obtained by applying the Bayesian inference from $\left\{ {y_{i} } \right\}_{BTS}$ is described as,

1 $$ p\left( {s{\text{|}}~\left\{ {y_{i} } \right\}_{{BTS}} ~} \right)~ \propto ~\int \left( {\mathop \prod \limits_{{i = 1}}^{m} \left( {A\left( {s,i} \right) \cdot exp \left( { - \frac{{\left( {y_{{iBTS}}  - f\left( {x{\text{|}}~s,i} \right)} \right)^{2} }}{{2\left( {\sigma _{{s,i}} } \right)^{2} }}} \right)} \right)} \right)dx $$

(see SI for details). Then, the input chemical species $s_{BTS}$ is estimated as the probability of species identification is maximised. The proportional expression (1) of $f\left( {x {|} s,i} \right)$ is a model function that describes relationships between the concentrations and index values of chemicals. Since no prior information about the relationships in *E. coli* was known, the model functions were determined by machine learning.

### Machine learning to deduce the chemotactic responses of *E. coli* cells

Numerical calculation of machine learning was achieved with the use of a custom-made programme based on Bayesian inference with MATLAB (Mathworks, Inc.) and performed by a computer (MAS-i7WX, TOWA ELECTRIC Co., Ltd.) equipped with a general-purpose graphics processing unit (Tesla K20). A training set was used for machine learning to increase the overall rate of accurate chemical identification.

## Supplementary Information

Supplementary Information.
