# Supplementary material for: Bayesian-based decipherment of in-depth information in bacterial chemical sensing beyond pleasant/unpleasant responses
Source: Sci Rep. 2022 Feb 22;12:2965. doi: 10.1038/s41598-022-06732-4 (PMC8863824; doi:10.1038/s41598-022-06732-4)
Supplement: Supplementary file 1 — Supplementary Information. [file 41598_2022_6732_MOESM1_ESM.pdf]

Supplementary information for

Bayesian-based decipherment of in-depth information in bacterial chemical sensing beyond pleasant/unpleasant responses

Hiroto Tanaka[1], Yasuaki Kazuta[1], Yasushi Naruse[2], Yukihiro Tominari[1], Hiroaki Umehara[2], Yoshiyuki Sowa[3], Takashi Sagawa[1], Kazuhiro Oiwa[1,2,4], Masato Okada[5], Ikuro Kawagishi[3*], Hiroaki Kojima[1*]

[1] Advanced ICT Research Institute, National Institute of Information and Communications Technology (NICT), Kobe, Hyogo 651-2492, Japan.

[2] Center for Information and Neural Networks (CiNet), National Institute of Information and Communications Technology (NICT) and Osaka University, Kobe, Hyogo 651-2492, Japan

[3] Department of Frontier Bioscience and Research Center for Micro-Nano Technology, Hosei University, Tokyo 184-8584, Japan

[4] Graduate School of Life Science, University of Hyogo, Harima Science Park City, Hyogo 678-1297, Japan

[5] Graduate School of Frontier Sciences, The University of Tokyo, Kashiwa 277-8561, Japan

*Correspondence to:
Dr. Hiroaki Kojima
Advanced ICT Research Institute
National Institute of Information and Communications Technology
Email: kojima@nict.go.jp
Phone: +81-78-969-2231
Fax: +81-78-969-2239

Dr. Ikuro Kawagishi
Department of Frontier Bioscience and Research Center for Micro-Nano Technology
Hosei University
Email: ikurok@hosei.ac.jp
Phone: +81-42-387-6235
Fax: +81-42-387-7002

Supplementary Information

Chemotaxis of cells of *Escherichia coli* (*E. coli*),

Chemotaxis of *E. coli* is swimming movement toward attractants or away from repellents. In the chemotaxis of *E. coli*, multiple receptors coupled to a single species of histidine kinase (named CheA) are expressed in a single cell equipped with locomotive actuators[1], i.e. flagella (Fig. S1a). Such an integrated unicellular signal transduction results in clockwise (CW) or counter clockwise (CCW) biased rotations of flagella, which correspond to run or tumble of swimming of cells. Note that attractant detachment schemes are shown in Fig. S1 for explanation, although we observed attractant attachment processes in this report. All six standard amino acids used in this report are attractant chemicals, and mainly activate Tar or Tsr receptors. Activation of Tar and/or Tsr receptors caused attractant responses (Fig. 1c and d in main text).

Adaptation of Chemotaxis of cells of *E. coli*.

E. coli cells show adaptation of chemotaxis for steady stimuli to dynamically modulate range of response, and to respond chemical gradient efficiently[2,3]. The adaptation is controlled by methylation level of the receptors[5] (as an example, Tar is shown in Fig. S1b). In case of adaptation for attractants, binding of attractants to receptors deactivates demethylase (CheB-P to CheB in Fig. S1b). Then, methylation level of receptors increases, and this results in increase of the amount of active CheA-P against the same concentrations of stimuli. Therefore, CW rotation increases, and cells adapt to persisting stimuli.

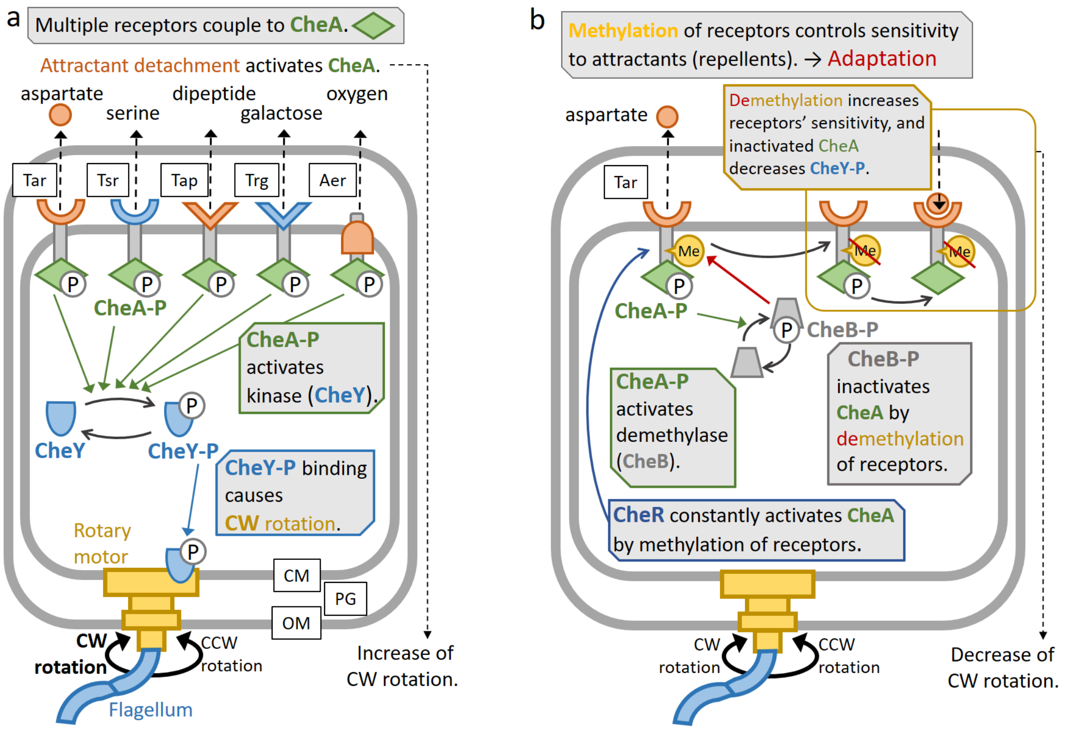

Figure S1 Chemotaxis and adaptation of *E. coli*.

a, Schematic drawing of main stream of signal transduction of chemotaxis in *E. coli*. Cells of *E. coli* have 5 types of receptors coupled to CheA, and respond to chemical stimuli (chemotaxis). Aspartate, serine, dipeptide, galactose and oxygen are typical attractants for Tar, Tsr, Tap, Trg and Aer receptors, respectively. Attractant detachment activates kinase, CheA, via phosphorylation. Phosphorylated CheA (CheA-P) phosphorylates CheY. Binding of phosphorylated CheY (CheY-P) biased direction of rotary motors in clockwise (CW). Oppositely, attractant attachment to receptors biased counter clockwise (CCW) rotations of motors. CM (cytoplasmic membrane), PG (peptidoglycan) and OM (outer membrane), P (phosphorylation of kinase). **b**, Schematic drawing of main stream of adaptation of chemotaxis. As an example and for explanation, we show the case of Tar receptor, and the case of attractant detachment. Initially, attractant detachment biases CW rotation of rotary motor, as a result of CheA-P activation (Fig. 1a). CheA-P simultaneously phosphorylates CheB to CheB-P, which is demethylase for receptors. Demethylation of receptors increases sensitivities of receptors, then affinities of receptors for attractants increase. As a result of increase of sensitivities of receptors, increase of amount of receptors binding attractants recovers CCW rotation.

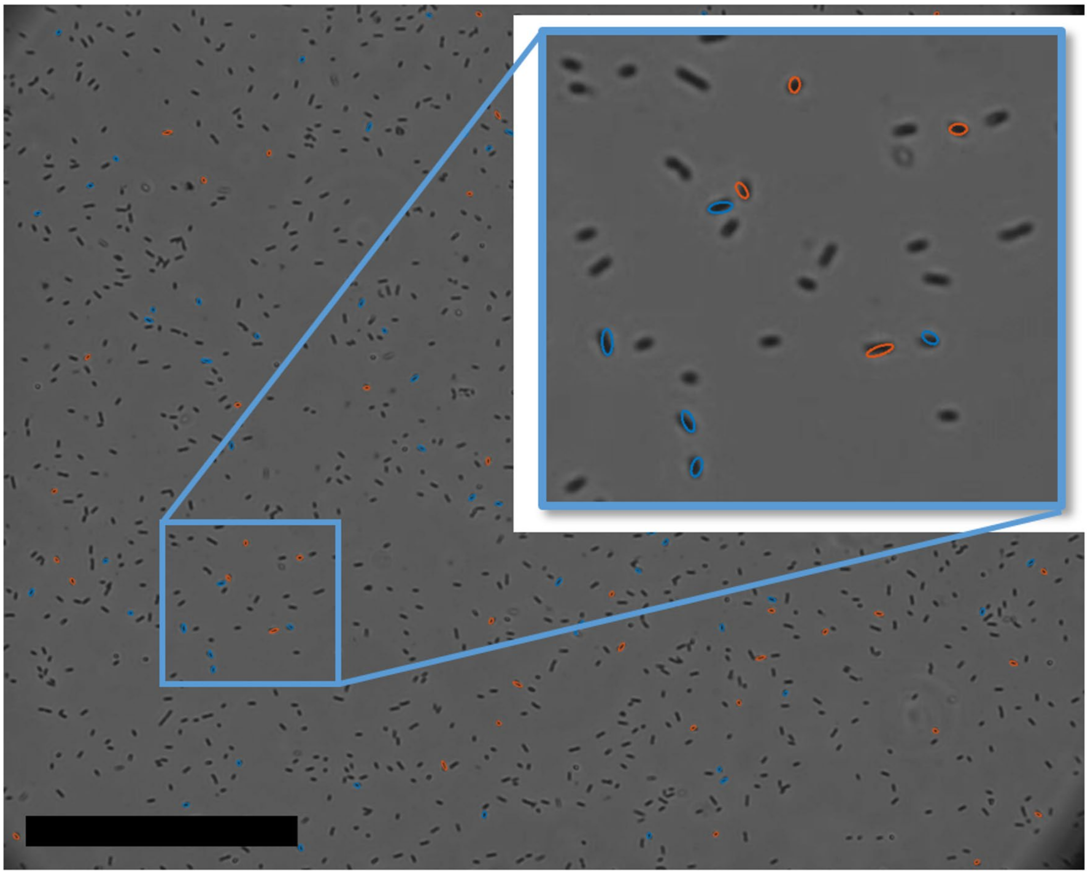

Figure S2. A phase-contrast image of tethered cells during observation and analysis of rotational motion.

Each black filled ellipse shows individual cells. Open colored ellipses show positions of moving cells after 1 frame (10 msec), and red and blue ellipses show CCW and CW rotations, respectively. The rotational motions of cells are analyzed by a custom-made program, which counts rotation angles of ellipses via binary images processed by adapted thresholds with OpenCV.

We counted rotational motions more than 7.5 degrees/frame[4] (see legend of Fig. 2c in main text). Note that our criteria for rotational displacement of > 7.5 degrees/frame were set not to accept rotational displacements of noise, which include thermal fluctuations and/or image processing errors, then, all rotational displacements < 7.5 degrees/frame were rejected. Scale bar is 100 μm.

Dimensional outline of microchannel.

In order to measure rotational motion during whole time range (with solution exchange flow), we used a PDMS (polydimethylsiloxane) microchannel device. We designed the micro channel to reduce flow speed[6], because physical perturbation of rotational motions of cell bodies by high flow speed disturbs output CW bias data. The disturb decreases reproducibility of data[7], and becomes source of error for statistical analysis. We had developed the design of micro channels by trial and error for supplying stable observation of rotational motion through solution exchange. With our microchannel devices (Fig. S3), we achieved stable measurements of whole CW bias outputs (during 10 minutes; Fig. 1c, d in main text).

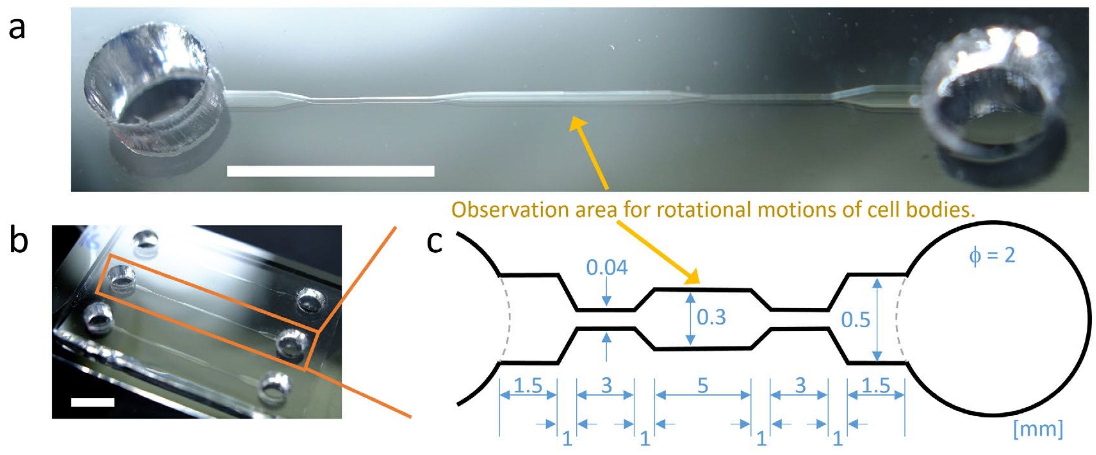

Figure S3. Design of a microchannel for quantitative measurement of whole process of rotational motion of tethered cells of *E. coli*.

a, An expanded photograph of one micro channel indicated by a rectangle in Fig. S3b. Two holes at both end of the channel are inlet and outlet of solution. Scale bar is 5 mm. **b,** Whole image of a PDMS (polydimethylsiloxane) microchannel on a coverslip. Scale bar is 5 mm. Chemotactic responses of cells (output) to a step input (in high temporal resolution; ramp input) were observed at yellow arrows indicated wide area, of which both sides are narrowed for reduction of flow speed. Schematic drawing of observation is shown in Fig. 1b in main text. **c,** engineering drawing of one microchannel. Both narrow areas of 0.04 mm width work to decrease flow speed.

Feature extraction from output responses (vectorization of outputs).

In order to handle output responses for computer calculations, it is necessary to extract features from the analog time course data of CW bias (Fig. 1d and 2a in main text, Fig. S4) and to quantify the features. When we look at the time courses of output responses just geometrically without biological knowledge (Fig. 1d, 2a in main text, and Fig. S4), we notice intuitively that they can be roughly fitted by six lines (time domains) as shown in Fig. S4. The lines correspond to (L1) initial flat area, (L2) CW bias ≈ 0 area that begins immediately after input stimulus (indicated black arrow in Fig. S4), (3) CW bias increase (recovery) area (including an overshoot part), (4) top flat CW bias area, (5) overshoot amount reduction area, (6) final flat area, respectively. Although it may be possible to characterize biological meaning for these six lines, here we dare to treat these lines as a template describing geometrical properties. We determined the fitting lines by adjusting size of lines with minimizing residual errors on the premise of template consisting of six lines. Then, we describe each output response numerically by an index set, which consists of 15 index values, $\{y_1, y_2, \ldots, y_{15}\}$. The index values are not necessary to refer to any processes of biological reactions in our present method, then they are arbitrarily set from appearance of output response waveform (Fig. S4). Here, we haven't hardly optimized setting of indexes, because our purpose of present study is construction of general procedure to apply Bayesian-based decipherment to microorganisms (*E. coli*). Optimization of the index set is our future plan at this stage. For making an index set, we use (1) to (6) lines, here. An output response is fit with 4 flat lines ((1), (2), (4), (6)) and 2 slope lines ((3), (5)). These 6 lines are defined by 9 fitting parameters. Although a set of 9 fitting parameters is sufficient to describe an output response, it is difficult to consider intuitively output response by only these 9 fitting parameters. Then we convert these 9 parameters to 15 index values, which allows us to interpret easily output response. An index set is defined by $\{y_1, y_2, \ldots, y_{15}\}$, where

y_1 : duration of line of area (2),
y_2 : amplitude of line of area (3),
y_3 : slope of line of area (3),
y_4 : duration of line of area (3),
y_5 : duration of line of area (4),
y_6 : minus amplitude of line of area (5),
y_7 : slope of line of area (5),
y_8 : duration of line of area (5),
y_9 : bias of line of area (6).

These 9 base index values correspond to fitting parameters of 6 lines. In addition, in order to interpret individual index set easily, we prepared additional indexes, which are,

y_{10} : bias of line of area (4),
y_{11} : bias of line of area (6) relative to area (2),
y_{12} : summation of duration of area (2) and (3),
y_{13} : summation of duration of area (3) and (5),
y_{14} : summation of duration of area (2) and (5),
y_{15} : summation of duration of area (2), (3) and (5).

Among these 15 index values, there are some indexes including same sources (duplicated values). Since we don't know which indexes are effective in this stage, we define an index set as a vector of 15 index values mentioned above. With this digitize procedure and with simple index values, a single output response can be expressed by an index set, $\{y_i\} = \{y_1, y_2, \dots, y_{15}\}$.

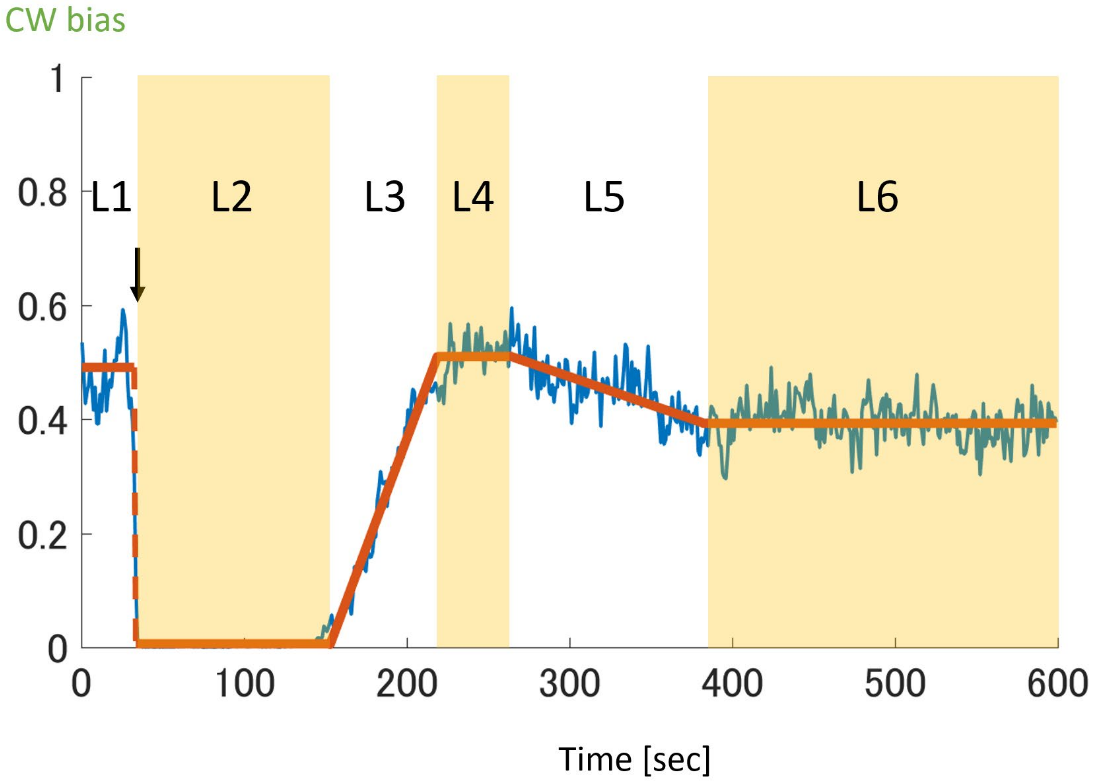

Figure S4

Feature extraction from output responses. We fitted individual 600 sec time courses by 6 lines. Each line corresponds to time domain of (1) to (6), respectively. Black arrow shows stimulation by addition of buffers including an attractant (L-glutamate (L-Glu), 3 mM). The 6 lines (areas) are divided just geometrically without biological knowledge, and fitted with 4 flat and 2 slope lines (see supplemental text for details). Finally, a single output response can be expressed by an index set, $\{y_i\} = \{y_1, y_2, \ldots, y_{15}\}$.

Identification of molecular species of chemical inputs based on Baysian inference.

To identify molecular species of chemical inputs based on Baysian inference, we relate the biological responses (for example, Figs. S4 and 1d in main text) to input chemical stimuli (signals), which are given as standard signals. Here, n species of input chemical stimuli (signals) are denoted by the symbol, $s = 1, 2, \dots, n$, and the input chemical stimuli consist of two parameters of chemical species, s, and their concentration, x. We prepared the index sets of $\{y_i\}$ to input chemical stimuli of (s, x). An index set of $\{y_i\}$ is generally assumed to be represented by arbitrary m of index values (here, we use 15 index values for one index set (vector), Figs. S4 and 2b in main text).

Here, we show algorithm to identify individual blind test samples (BTS) from each observed {y_(i_BTS) } which is response to BTS input (s_BTS,x_BTS), where input chemicals indicated with s_BTS (= 1,2,…,n). The identification is performed by estimating $p(s \mid \{y_i\})$ for the number of s statistically through the following mathematical steps.

The aim of this mathematical derivation is to obtain probability function of $p(s \mid \{y_i\})$, that is, to estimate probability of s from the observed individual index set $\{y_i\}$. First, by using Bayes' theorem, $p(s, x \mid \{y_i\})$ is described as,

$$p(s, x \mid \{y_i\}) = \frac{p(\{y_i\} \mid s,x) \cdot p(s,x)}{p(\{y_i\})}. \qquad \text{(s1)}$$

Equation (s1) is described as,

$$p(s, x \mid \{y_i\}) \propto p(\{y_i\} \mid s, x), \qquad \text{(s2)}$$

because $p(s, x)$ and $p(\{y_i\})$ are constant, here. Probability function $p(\{y_i\} \mid s, x)$ of proportional expression (s2) describes probability of observation for an index set $\{y_i\}$ under the condition of input stimulus of chemical species of s and its concentration of x. The $p(\{y_i\} \mid s, x)$ is described as,

$$p(\{y_i\} \mid s, x) \propto \prod_{i=1}^{m} \left(exp \left(-\frac{\left(y_i - f_{k_i}(x \mid s, i)\right)^2}{2\left(\sigma_{s,i}\right)^2} \right) \right), \qquad \text{(s3)}$$

where, $f_{k_i}(x \mid s, i)$ and $\sigma_{s,i}$ are model function and standard deviation, respectively, and both are determined for each (s, i). The model function $f_{k_i}(x \mid s, i)$ is a function of x,

representing relationship between each index value y_i and x under the condition of chemical stimulus, s. The model functions are determined by machine learning by using training data. Here, we select one of 1st – 4th order functions as a model function for each index value by using machine learning as mentioned in next section. The individual standard deviation $\sigma_{s,i}$ is calculated by index values $y_{s,i}$ from observed data (training data) and the individual model function. We introduce proportional expression (s3) to (s2), then,

$$p(s, x \mid \{y_i\}) \propto \prod_{i=1}^{m} \left(exp\left(-\frac{\left(y_i - f_{k_i}(x \mid s, i)\right)^2}{2\left(\sigma_{s,i}\right)^2} \right) \right). \qquad \text{(s4)}$$

In order to compare proportional expression (s4) among different species of s, we normalize proportional expression (s4), then,

$$p(s\ , x \mid \{y_i\}) \propto \prod_{i=1}^{m} \left(A(s,i) \cdot exp\left(-\frac{\left(y_i - f_{k_i}(x \mid s, i)\right)^2}{2\left(\sigma_{s,i}\right)^2} \right) \right), \text{(s5)}$$

where, each normalized coefficient $A(s,i)$ is calculated as,

$$A(s,i) = \frac{1}{\int \left(exp\left(-\frac{\left(y_i - f_{k_i}(x \mid s, i)\right)^2}{2\left(\sigma_{s,i}\right)^2} \right) \right) dx}.$$

Then, probability function $p(s \mid \{y_i\})$, representing probability of input stimulus of s under the observed $\{y_i\}$ condition is described as,

$$p(s \mid \{y_i\}) \propto \int \left(\prod_{i=1}^{m} \left(A(s,i) \cdot exp\left(-\frac{\left(y_i - f_{k_i}(x \mid s, i)\right)^2}{2\left(\sigma_{s,i}\right)^2} \right) \right) \right) dx. \qquad \text{(s6)}$$

For estimation of input stimulus, we calculate $p(s \mid \{y_i\})$ for each $s(= 1, 2, \dots, n)$, and we select the highest $p(s \mid \{y_i\})$ as estimated input $\hat{s}$.

Machine learning for model function.

We use "model function $f_{k_i}(x \mid s, i)$", which relates input stimulus (s, i) to observable index sets $\{y_i\}$, for identification of attractants in Bayesian inference formulation (s6) in this study. However, it is not obvious what kind of function is appropriate for identification of attractants in this stage. Therefore, we prepare simple 1st – 4th order functions as candidates for model function for each index value, and choose one of them as optimal function by using machine learning with leave-one-out cross validation. It is not obvious whether chosen function is the most optimal function or not in this stage, but we use the simple model functions, because our purpose of present study is principle proof and complex functions needs high computer power and long calculation time. Then, we prepare,

$$
\begin{aligned}
f_4(x \mid s, i) &= \mathrm{a}_{s,i} \cdot x^4 + \mathrm{b}_{s,i} \cdot x^3 + \mathrm{c}_{s,i} \cdot x^2 + \mathrm{d}_{s,i} \cdot x + \mathrm{e}_{s,i} \ , \\
f_3(x \mid s, i) &= \mathrm{b}_{s,i} \cdot x^3 + \mathrm{c}_{s,i} \cdot x^2 + \mathrm{d}_{s,i} \cdot x + \mathrm{e}_{s,i} \ , \\
f_2(x \mid s, i) &= \mathrm{c}_{s,i} \cdot x^2 + \mathrm{d}_{s,i} \cdot x + \mathrm{e}_{s,i} \ , \\
f_1(x \mid s, i) &= \mathrm{d}_{s,i} \cdot x + \mathrm{e}_{s,i} \ ,
\end{aligned}
$$

as candidates for model functions. We carried out Bayesian inference in mathematical constraint condition of only four candidate functions of $f_{k_i}(x \mid s, i)$ for model functions, and determined the coefficients and optimum order of model functions (Fig. S5). The coefficients $(\mathrm{a}_{s,i}, \mathrm{b}_{s,i}, \mathrm{c}_{s,i}, \mathrm{d}_{s,i}, \mathrm{e}_{s,i})$ are determined by fitting to observed index sets $\{y_{s,i}\}$ (training data), and the optimum orders (k_i) are determined by machine learning with leave-one-out cross validation.

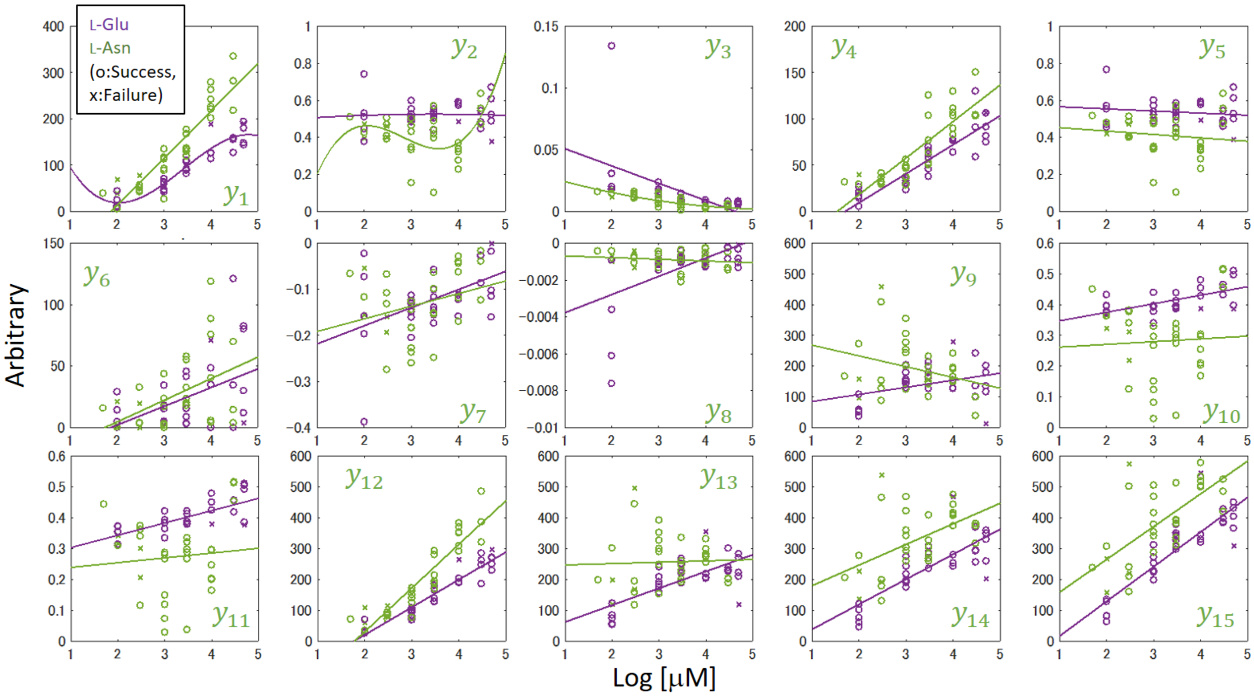

Figure S5

Concentration dependencies of indexes $\{y_i\}$ of characteristic vectors obtained with L-glutamate (L-Glu; purple) and L-asparagine (L-Asn; green). Each graph is coloured according to chemical species same as Fig. 2c. Plot makers of 'o' indicate data that succeed to identify blind samples, and 'x' fails to identify. Solid lines show model functions representing concentration dependencies. Note that the identification accuracies and model functions were evaluated with leave-one-out cross validation.

Calculation of standard deviation of accuracy rates for blind data sets under the condition of random selection.

Here, we consider probability functions (PF) of number of correct answer ($\hat{s} = s_{BTS}$), $k_{total} = \sum_{j=1}^{N}(k_j)$, under the condition that total number of blind test samples is $n_{total} = \sum_{j=1}^{N}(n_j)$, where n_j and k_j are number of BTS data and correct answer for individual chemicals tagged with j, respectively.

In case of groups consisting of 2 chemicals ($N = 2$), individual PFs of number of correct answer for each chemical, $p_{c1}(k_1|n_1, n_2)$ and $p_{c2}(k_2|n_1, n_2)$ are described by binomial distribution as,

$$p_{c1}(k_1|n_1, n_2) = {}_{n_1}\mathrm{C}_{k_1} \cdot f_1^{\,k_1} \cdot f_2^{\,n_1-k_1},$$
$$p_{c2}(k_2|n_1, n_2) = {}_{n_2}\mathrm{C}_{k_2} \cdot f_2^{\,k_2} \cdot f_1^{\,n_2-k_2}.$$

Here, constraint conditions are $n_{total} = n_1 + n_2$ and $k_{total} = k_1 + k_2$. ($0 \le k_1 \le n_1,\ 0 \le k_2 \le n_2$), and we introduce fractions of $f_1 = \frac{n_1}{n_{total}}$ and $f_2 = \frac{n_2}{n_{total}}$. Then, PF, $p(k_{total}|n_1, n_2)$, is described as,

$$p(k_{total}|n_1, n_2) = \sum_{k_1=0}^{\min(n_1, k_{total})}\left(p_{c1}(k_1|n_1, n_2) \cdot p_{c2}(k_{total} - k_1|n_1, n_2)\right),$$

and we calculated PFs under the condition of random selection for each group (Fig. 2d, 4c, S8a).

In case of groups consisting of 3 chemicals ($N = 3$), individual PFs of number of correct answer for each chemical, $p_{cj}(k_j|n_1, n_2, n_3)$ are described based on binomial distribution as,

$$p_{c1}(k_1|n_1, n_2, n_3) = {}_{n_1}\mathrm{C}_{k_1} \cdot f_1^{\,k_1} \cdot \sum_{l_1=0}^{n_1-k_1}\left({}_{n_1-k_1}\mathrm{C}_{l_1} \cdot f_2^{\,l_1} \cdot f_3^{\,n_1-k_1-l_1}\right),$$
$$p_{c2}(k_2|n_1, n_2, n_3) = {}_{n_2}\mathrm{C}_{k_2} \cdot f_2^{\,k_2} \cdot \sum_{l_2=0}^{n_2-k_2}\left({}_{n_2-k_2}\mathrm{C}_{l_2} \cdot f_3^{\,l_2} \cdot f_1^{\,n_2-k_2-l_2}\right),$$
$$p_{c3}(k_3|n_1, n_2, n_3) = {}_{n_3}\mathrm{C}_{k_3} \cdot f_3^{\,k_3} \cdot \sum_{l_3=0}^{n_3-k_3}\left({}_{n_3-k_3}\mathrm{C}_{l_3} \cdot f_1^{\,l_3} \cdot f_2^{\,n_3-k_3-l_3}\right),$$

where $f_j = \frac{n_j}{n_{total}}$.

Finally, PF, $p(k_{total}|n_1, n_2, n_3)$ is described as,

$$p(k_{total}|n_1, n_2, n_3) = \sum_{k_1=0}^{\min(n_1, k_{total})}\left\{p_{c1}(k_1|n_1, n_2, n_3) \cdot \sum_{k_2=0}^{\min(n_2, (k_{total}-k_1))}\left(p_{c2}(k_2|n_1, n_2, n_3) \cdot p_{c3}(k_{total} - k_1 - k_2|n_1, n_2, n_3)\right)\right\},$$

where $n_{total} = n_1 + n_2 + n_3$ and $k_{total} = k_1 + k_2 + k_3$. ($0 \le k_1 \le n_1$, $0 \le k_2 \le n_2$, $0 \le k_3 \le n_3$) (Fig.S8b).

In case of groups consisting of 4, 5, 6 chemicals ($N = 4,5,6$), with $p_{cj}(k_j|\{n_i\})$, PFs, $p(k_{total}|\{n_i\})$ are described as,

$$p(k_{total}|n_1,n_2,n_3,n_4) = \sum_{k_1=0}^{\min(n_1,k_{total})}\left[p_{c1}(k_1|\{n_i\})\cdot\sum_{k_2=0}^{\min(n_2,(k_{total}-k_1))}\left\{p_{c2}(k_2|\{n_i\})\cdot\sum_{k_3=0}^{\min(n_3,(k_{total}-k_1-k_2))}\left(p_{c3}(k_3|\{n_i\})\cdot p_{c4}(k_{total}-k_1-k_2-k_3|\{n_i\})\right)\right\}\right],$$

$$p(k_{total}|n_1,n_2,n_3,n_4,n_5) = \sum_{k_1=0}^{\min(n_1,k_{total})}\left(p_{c1}(k_1|\{n_i\})\cdot\sum_{k_2=0}^{\min(n_2,(k_{total}-k_1))}\left(p_{c2}(k_2|\{n_i\})\cdot\sum_{k_3=0}^{\min(n_3,(k_{total}-k_1-k_2))}\left(p_{c3}(k_3|\{n_i\})\cdot\sum_{k_4=0}^{\min(n_4,(k_{total}-k_1-k_2-k_3))}\left(p_{c4}(k_4|\{n_i\})\cdot p_{c5}(k_{total}-k_1-k_2-k_3-k_4|\{n_i\})\right)\right)\right)\right),$$

$$p(k_{total}|n_1,n_2,n_3,n_4,n_5,n_6) = \sum_{k_1=0}^{\min(n_1,k_{total})}\left(p_{c1}(k_1|\{n_i\})\cdot\sum_{k_2=0}^{\min(n_2,(k_{total}-k_1))}\left(p_{c2}(k_2|\{n_i\})\cdot\sum_{k_3=0}^{\min(n_3,(k_{total}-k_1-k_2))}\left(p_{c3}(k_3|\{n_i\})\cdot\sum_{k_4=0}^{\min(n_4,(k_{total}-k_1-k_2-k_3))}\left(p_{c4}(k_4|\{n_i\})\cdot\sum_{k_5=0}^{\min(n_5,(k_{total}-k_1-k_2-k_3-k_4))}\left(p_{c5}(k_5|\{n_i\})\cdot p_{c6}(k_{total}-k_1-k_2-k_3-k_4-k_5|\{n_i\})\right)\right)\right)\right)\right),$$

respectively. We calculated $p(k_{total}|\{n_i\})$ numerically (Fig.3b, Fig.S8c,d,e).

Quantitative measurements of response outputs for 6 standard chemicals.

We prepared six amino acids as standard chemicals in this report. All are attractant chemicals for cells of *E. coli*, L-aspartate (L-Asp), L-glutamate (L-Glu), D-aspartate (D-Asp), L-asparagine (L-Asn), L-cysteine (L-Cys) and L-serine (L-Ser) [8]. For statistical treatments, we assigned substrate number, $s = 1, 2, \ldots, 6$, to individual chemicals, 1. L-Asp, 2. L-Glu, 3. D-Asp, 4. L-Asn, 5. L-Cys and 6. L-Ser, respectively. Figures S6 and S7 show gallery of typical output responses (CW biases) and concentration dependencies of 15 indexes, respectively.

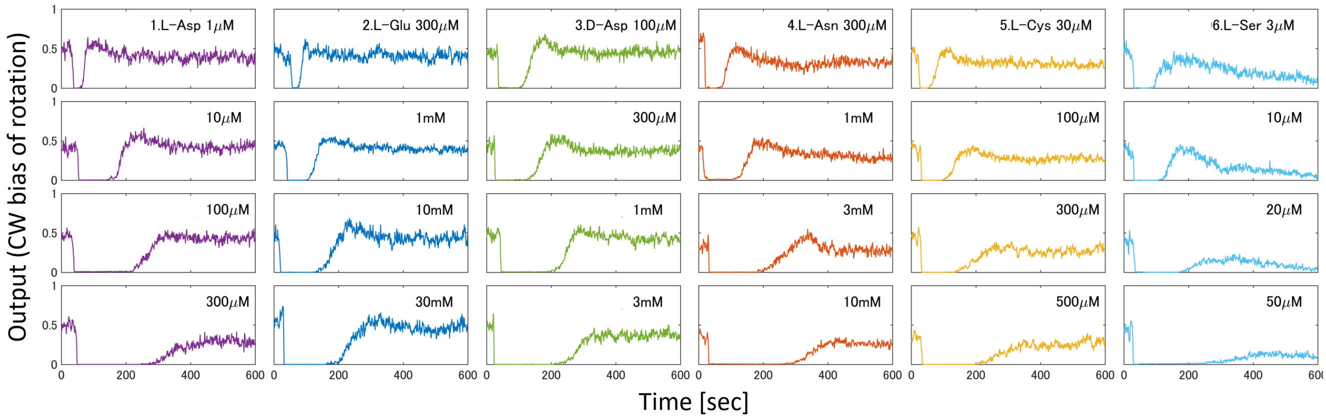

Figure S6 Gallery of typical outputs 6 standard chemicals.

Each graph is colored according to chemical species (purple; 1. L-Asp, blue; 2. L-Glu, green; 3. D-Asp, red; 4. L-Asn, yellow; 5. L-Cys, light blue; 6. L-Ser). Regardless of chemical species, higher concentrations of attractants cause longer attractant responses. Although concentration dependencies of CW biases are different due to chemical species, geometric profiles are similar.

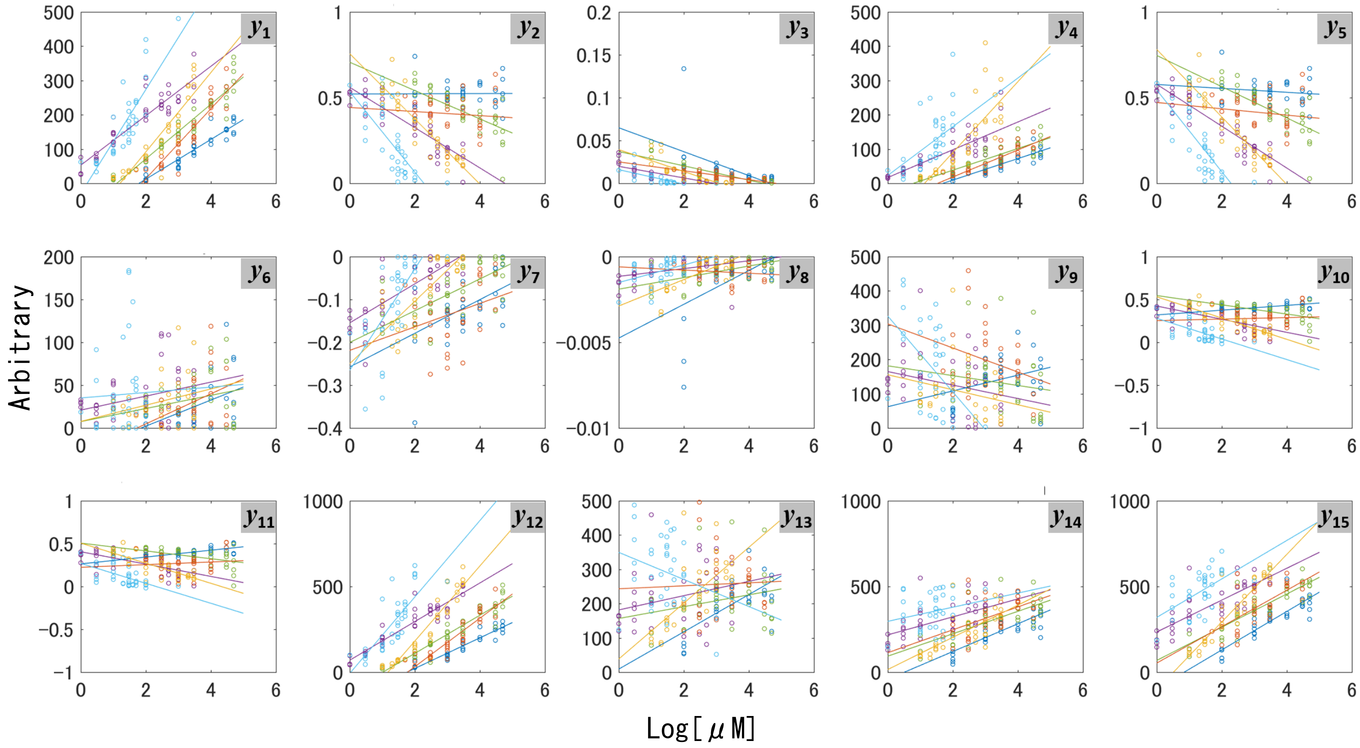

Figure S7

Gallery of concentration dependences of index sets. Index sets were calculated from output traces. Horizontal axes indicate concentrations of chemicals (attractants) in log scale, and vertical axes indicate index values. Markers in graphs are colored according to chemical species same as Fig.S6. These graphs show dependences of index sets (15 index values of individual characteristic vectors) on both attractant types and concentrations. Each line in graphs shows leaner model function for representing a dependence of index value to concentrations. We use differences of dependences of index values to concentrations to identify input blind samples. Then, with these standard index sets, by using Bayesian inference with machine learning, we succeeded to construct DeSIRAM. Accuracy rate of decipherment of chemical types of blind samples with linear model functions among these six amino acids is 32 % (N = 210, middle bar in Fig. S8d). Simple linear model function seems to represent dependences of index values to concentration insufficiently. In fact, by using high order functions as model functions, identification rate among these six amino acids improved to be 49 % (Table S3).

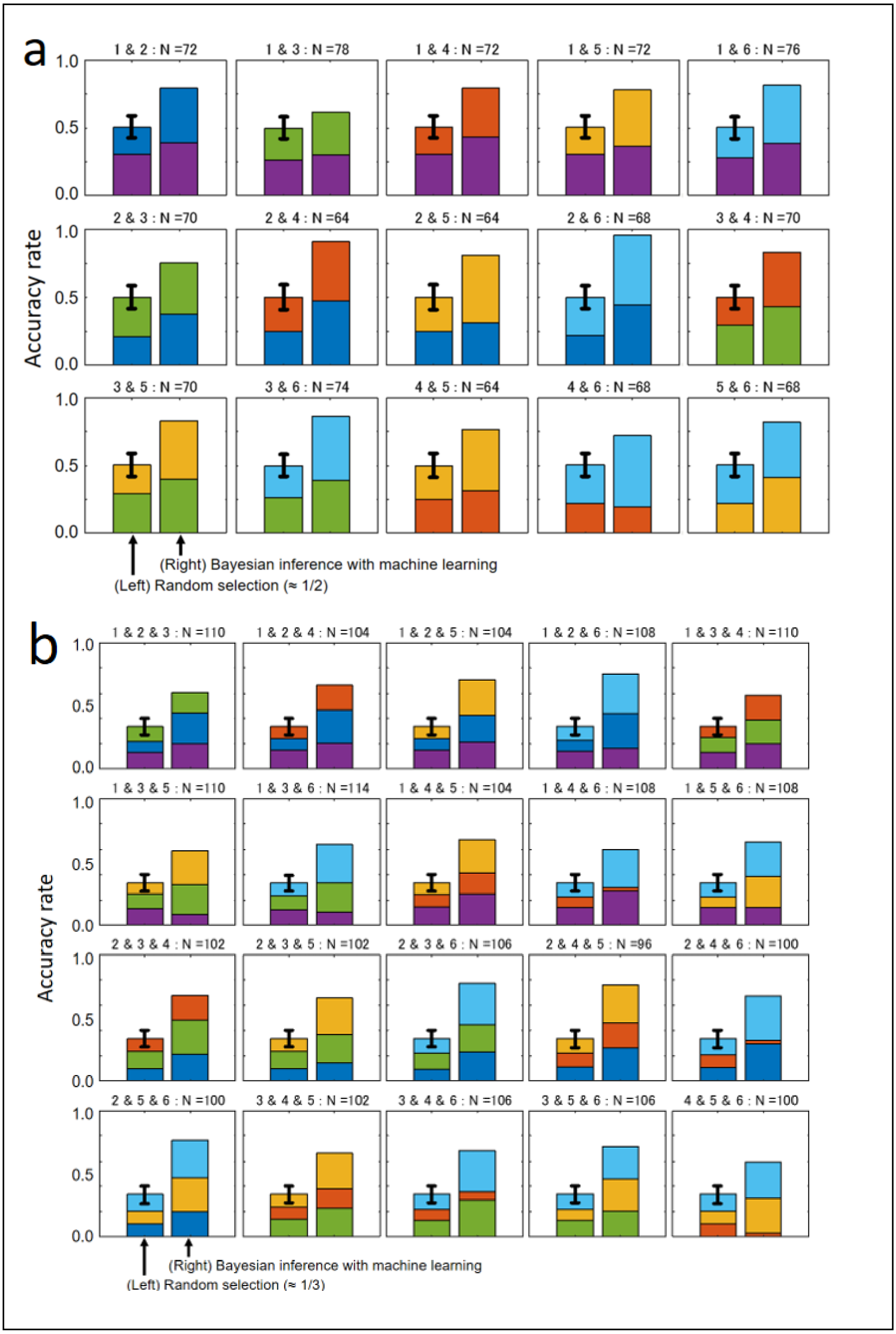
a
b
Accuracy rate
1 & 2 : N =72
1 & 3 : N =78
1 & 4 : N =72
1 & 5 : N =72
1 & 6 : N =76
2 & 3 : N =70
2 & 4 : N =64
2 & 5 : N =64
2 & 6 : N =68
3 & 4 : N =70
3 & 5 : N =70
3 & 6 : N =74
4 & 5 : N =64
4 & 6 : N =68
5 & 6 : N =68
(Right) Bayesian inference with machine learning
(Left) Random selection (≈ 1/2)
1 & 2 & 3 : N =110
1 & 2 & 4 : N =104
1 & 2 & 5 : N =104
1 & 2 & 6 : N =108
1 & 3 & 4 : N =110
1 & 3 & 5 : N =110
1 & 3 & 6 : N =114
1 & 4 & 5 : N =104
1 & 4 & 6 : N =108
1 & 5 & 6 : N =108
2 & 3 & 4 : N =102
2 & 3 & 5 : N =102
2 & 3 & 6 : N =106
2 & 4 & 5 : N =96
2 & 4 & 6 : N =100
2 & 5 & 6 : N =100
3 & 4 & 5 : N =102
3 & 4 & 6 : N =106
3 & 5 & 6 : N =106
4 & 5 & 6 : N =100
(Right) Bayesian inference with machine learning
(Left) Random selection (≈ 1/3)

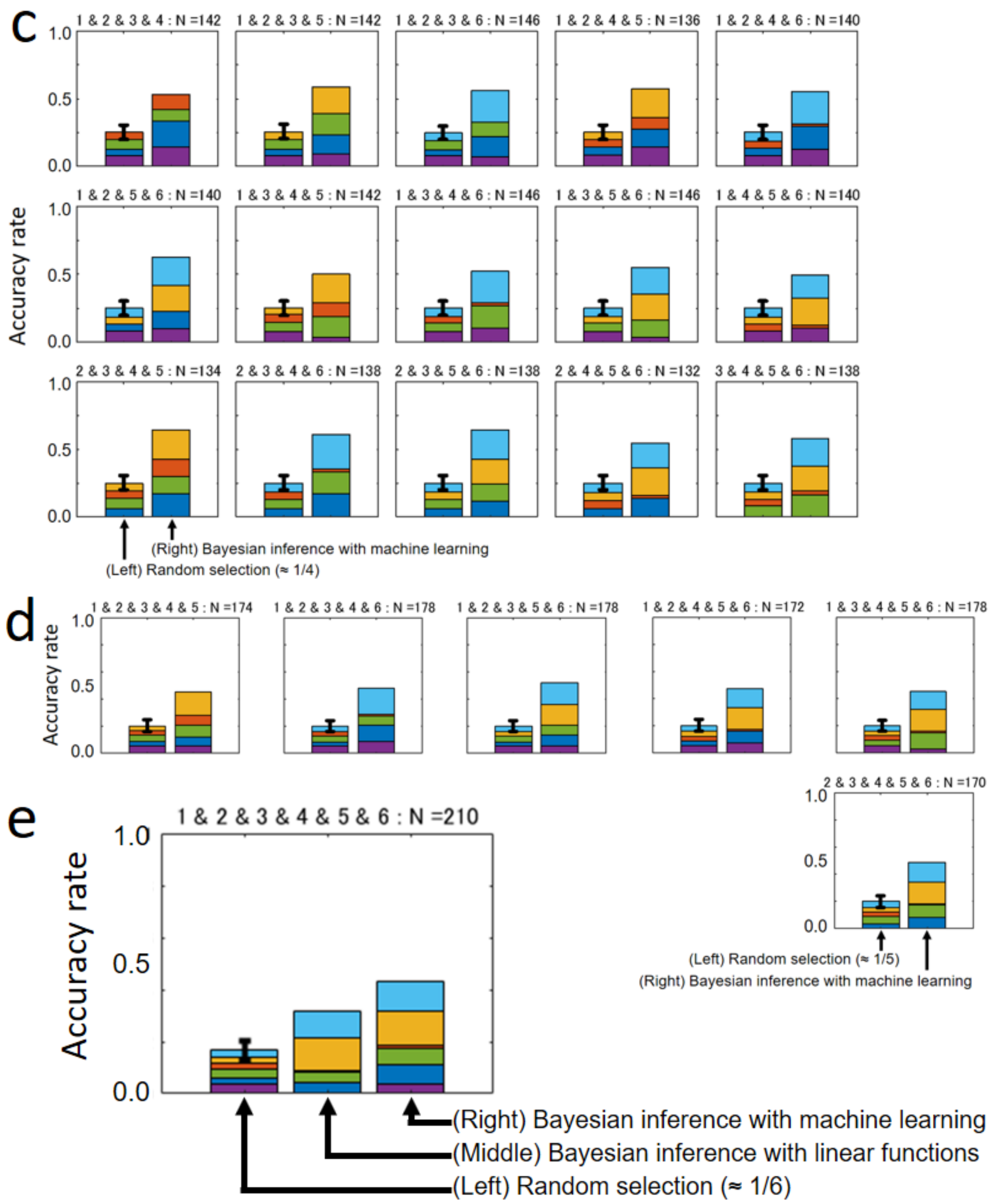

Figure S8
Gallery of identification accuracies for two (a), three (b), four (c), five (d) and six (e) chemical attractants groups. Tag numbers of including chemical compounds and total number of data are indicated upside of each graph. Tag number and colors are same as Fig. S6. Left bar in each graph shows theoretical identification accuracy under the condition of random selection. Black line on left bar shows standard deviation calculated numerically. Right bars show accuracy rates by Bayesian inference with machine learning by using 1st-4th order functions. Middle bar in Fig. S8e shows an accuracy rate by Bayesian inference with linear functions $(f_1(x \mid s, i))$. All accuracy rates by our method exceed those by random selection, although accuracy rates seem to depend on combinations of chemicals.

Table S1. Comparison of identification accuracies of DeSIRAM to those of random selection (RS). Case of groups consisting of 2 chemicals.

	Results by RS	Results by DeSIRAM		Probability of occurrence of numbers by DeSIRAM under RS
2 chemical samples	Calculated number of success (mean±SD)	Number of success	Accuracy (success / total)	
1.L-Asp, 2.L-Glu (N = 40, 32)	36.4 ± 6.0	57	0.79	3.6×10^{-7}
1.L-Asp, 3.D-Asp (N = 40, 38)	39.0 ± 6.3	48	0.62	1.2×10^{-2}
1.L-Asp, 4.L-Asn (N = 40, 32)	36.4 ± 6.0	57	0.79	3.6×10^{-7}
1.L-Asp, 5.L-Cys (N = 40, 32)	36.4 ± 6.0	56	0.78	1.3×10^{-6}
1.L-Asp, 6.L-Ser (N = 40, 36)	38.1 ± 6.2	62	0.82	1.0×10^{-8}
2.L-Glu, 3.D-Asp (N = 32, 38)	35.3 ± 5.9	53	0.76	8.1×10^{-6}
2.L-Glu, 4.L-Asn (N = 32, 32)	32.0 ± 5.7	58	0.91	4.1×10^{-12}
2.L-Glu, 5.L-Cys (N = 32, 32)	32.0 ± 5.7	52	0.81	1.8×10^{-7}
2.L-Glu, 6.L-Ser (N = 32, 36)	34.1 ± 5.8	65	0.96	1.9×10^{-16}
3.D-Asp, 4.L-Asn (N = 38, 32)	35.3 ± 5.9	58	0.83	1.1×10^{-8}
3.D-Asp, 5.L-Cys (N = 38, 32)	35.3 ± 5.9	58	0.83	1.1×10^{-8}
3.D-Asp, 6.L-Ser (N = 38, 36)	37.0 ± 6.1	64	0.86	3.9×10^{-11}
4.L-Asn, 5.L-Cys (N = 32, 32)	32.0 ± 5.7	49	0.77	8.7×10^{-6}
4.L-Asn, 6.L-Ser (N = 32, 36)	34.1 ± 5.8	49	0.72	1.2×10^{-4}
5.L-Cys, 6.L-Ser (N = 32, 36)	34.1 ± 5.8	56	0.82	2.7×10^{-8}

Table S2. Comparison of identification accuracies of DeSIRAM to those of random

selection (RS). Case of groups consisting of 3 chemicals.

	Results by RS	Results by DeSIRAM		Probability of occurrence of numbers by DeSIRAM under RS
3 chemical samples	Calculated number of success (mean±SD)	Number of success	Accuracy (success / total)	
1.L–Asp, 2.L–Glu, 3.D–Asp (N = 40, 32, 38)	36.9 ± 7.0	67	0.61	2.9×10^{-9}
1.L–Asp, 2.L–Glu, 4.L–Asn (N = 40, 32, 32)	35.0 ± 6.8	69	0.66	7.5×10^{-12}
1.L–Asp, 2.L–Glu, 5.L–Cys (N = 40, 32, 32)	35.0 ± 6.8	74	0.71	5.0×10^{-15}
1.L–Asp, 2.L–Glu, 6.L–Ser (N = 40, 32, 36)	36.2 ± 6.9	81	0.75	1.2×10^{-18}
1.L–Asp, 3.D–Asp, 4.L–Asn (N = 40, 38, 32)	36.9 ± 7.0	64	0.58	7.0×10^{-8}
1.L–Asp, 3.D–Asp, 5.L–Cys (N = 40, 38, 32)	36.9 ± 7.0	65	0.59	2.5×10^{-8}
1.L–Asp, 3.D–Asp, 6.L–Ser (N = 40, 38, 36)	38.0 ± 7.1	73	0.64	1.6×10^{-11}
1.L–Asp, 4.L–Asn, 5.L–Cys (N = 40, 32, 32)	35.0 ± 6.8	71	0.68	4.6×10^{-13}
1.L–Asp, 4.L–Asn, 6.L–Ser (N = 40, 32, 36)	36.2 ± 6.9	65	0.60	9.2×10^{-9}
1.L–Asp, 5.L–Cys, 6.L–Ser (N = 40, 32, 36)	36.2 ± 6.9	71	0.66	6.4×10^{-12}
2.L–Glu, 3.D–Asp, 4.L–Asn (N = 32, 38, 32)	34.2 ± 6.7	69	0.68	1.6×10^{-12}
2.L–Glu, 3.D–Asp, 5.L–Cys (N = 32, 38, 32)	34.2 ± 6.7	67	0.66	2.5×10^{-11}
2.L–Glu, 3.D–Asp, 6.L–Ser (N = 32, 38, 36)	35.4 ± 6.9	82	0.77	2.2×10^{-20}
2.L–Glu, 4.L–Asn, 5.L–Cys (N = 32, 32, 32)	31.9 ± 6.5	73	0.76	1.1×10^{-17}
2.L–Glu, 4.L–Asn, 6.L–Ser (N = 32, 32, 36)	33.4 ± 6.7	67	0.67	5.5×10^{-12}
2.L–Glu, 5.L–Cys, 6.L–Ser (N = 32, 32, 36)	33.4 ± 6.7	77	0.77	4.7×10^{-19}
3.D–Asp, 4.L–Asn, 5.L–Cys (N = 38, 32, 32)	34.2 ± 6.7	68	0.67	6.3×10^{-12}
3.D–Asp, 4.L–Asn, 6.L–Ser (N = 38, 32, 36)	35.4 ± 6.9	73	0.69	8.3×10^{-14}
3.D–Asp, 5.L–Cys, 6.L–Ser (N = 38, 32, 36)	35.4 ± 6.9	76	0.72	8.2×10^{-16}
4.L–Asn, 5.L–Cys, 6.L–Ser (N = 32, 32, 36)	33.4 ± 6.7	59	0.59	9.5×10^{-8}

Table S3. Comparison of identification accuracies of DeSIRAM to those of random selection (RS). Case of groups consisting of 4 chemicals.

	Results by RS	Results by DeSIRAM		Probability of occurrence of numbers by DeSIRAM under RS
4 chemical samples	Calculated number of success (mean±SD)	Number of success	Accuracy (success / total)	
1.L-Asp, 2.L-Glu, 3.D-Asp, 4.L-Asn (N = 40, 32, 38, 32)	35.7 ± 7.3	76	0.54	4.1×10^{-13}
1.L-Asp, 2.L-Glu, 3.D-Asp, 5.L-Cys (N = 40, 32, 38, 32)	37.1 ± 7.5	84	0.59	2.3×10^{-16}
1.L-Asp, 2.L-Glu, 3.D-Asp, 6.L-Ser (N = 40, 32, 38, 36)	36.6 ± 7.4	82	0.56	1.2×10^{-15}
1.L-Asp, 2.L-Glu, 4.L-Asn, 5.L-Cys (N = 40, 32, 32, 32)	34.2 ± 7.2	78	0.57	1.4×10^{-15}
1.L-Asp, 2.L-Glu, 4.L-Asn, 6.L-Ser (N = 40, 32, 32, 36)	35.2 ± 7.3	78	0.56	1.1×10^{-14}
1.L-Asp, 2.L-Glu, 5.L-Cys, 6.L-Ser (N = 40, 32, 32, 36)	35.2 ± 7.3	88	0.63	4.8×10^{-21}
1.L-Asp, 3.D-Asp, 4.L-Asn, 5.L-Cys (N = 40, 38, 32, 32)	35.7 ± 7.3	71	0.50	1.3×10^{-10}
1.L-Asp, 3.D-Asp, 4.L-Asn, 6.L-Ser (N = 40, 38, 32, 36)	36.6 ± 7.4	76	0.52	2.2×10^{-12}
1.L-Asp, 3.D-Asp, 5.L-Cys, 6.L-Ser (N = 40, 38, 32, 36)	36.6 ± 7.4	80	0.55	1.6×10^{-14}
1.L-Asp, 4.L-Asn, 5.L-Cys, 6.L-Ser (N = 40, 32, 32, 36)	35.2 ± 7.3	69	0.49	5.0×10^{-10}
2.L-Glu, 3.D-Asp, 4.L-Asn, 5.L-Cys (N = 32, 38, 32, 32)	33.6 ± 7.1	87	0.65	2.8×10^{-22}
2.L-Glu, 3.D-Asp, 4.L-Asn, 6.L-Ser (N = 32, 38, 32, 36)	34.6 ± 7.2	84	0.61	5.8×10^{-19}
2.L-Glu, 3.D-Asp, 5.L-Cys, 6.L-Ser (N = 32, 38, 32, 36)	34.6 ± 7.2	89	0.64	1.8×10^{-22}
2.L-Glu, 4.L-Asn,, 5.L-Cys, 6.L-Ser (N = 32, 32, 32, 36)	33.0 ± 7.0	72	0.55	3.5×10^{-13}
3.D-Asp, 4.L-Asn,, 5.L-Cys, 6.L-Ser (N = 38, 32, 32, 36)	34.6 ± 7.2	80	0.58	2.1×10^{-16}

Table S4. Comparison of identification accuracies of DeSIRAM to those of random selection (RS). Case of groups consisting of 5 chemicals.

	Results by RS	Results by DeSIRAM		Probability of occurrence of numbers by DeSIRAM under RS
5 chemical samples	Calculated number of success (mean ±SD)	Number of success	Accuracy (success / total)	
1.L-Asp, 2.L-Glu, 3.D-Asp, 4.L-Asn, 5.L-Cys (N = 40, 32, 38, 32, 32)	35.0 ± 7.5	79	0.45	4.2×10^{-14}
1.L-Asp, 2.L-Glu, 3.D-Asp, 4.L-Asn, 6.L-Ser (N = 40, 32, 38, 32, 36)	35.7 ± 7.6	85	0.48	1.1×10^{-16}
1.L-Asp, 2.L-Glu, 3.D-Asp, 5.L-Cys, 6.L-Ser (N = 40, 32, 38, 32, 36)	35.7 ± 7.6	93	0.52	1.7×10^{-21}
1.L-Asp, 2.L-Glu, 4.L-Asn, 5.L-Cys, 6.L-Ser (N = 40, 32, 32, 32, 36)	34.5 ± 7.4	81	0.47	1.5×10^{-15}
1.L-Asp, 3.D-Asp, 4.L-Asn, 5.L-Cys, 6.L-Ser (N = 40, 38, 32, 32, 36)	35.7 ± 7.6	81	0.46	1.6×10^{-14}
2.L-Glu, 3.D-Asp, 4.L-Asn, 5.L-Cys, 6.L-Ser (N = 32, 38, 32, 32, 36)	34.0 ± 7.4	83	0.49	4.1×10^{-17}

Table S5. Comparison of identification accuracies of DeSIRAM to those of random selection (RS). Case of groups consisting of 5 chemicals.

	Results by RS	Results by DeSIRAM		Probability of occurrence of numbers by DeSIRAM under RS
6 chemical samples	Calculated number of success (mean ±SD)	Number of success	Accuracy (success / total)	
1.L-Asp, 2.L-Glu, 3.D-Asp, 4.L-Asn, 5.L-Cys, 6.L-Ser (N = 40, 32, 38, 32, 32, 36)	35.1 ± 7.7	91	0.43	1.3×10^{-19}

REFERENCES (SI)

1 Parkinson, J. S., Hazelbauer, G. L. & Falke, J. J. Signaling and sensory adaptation in Escherichia coli chemoreceptors: 2015 update. *Trends in microbiology* **23**, 257-266, doi:10.1016/j.tim.2015.03.003 (2015).

2 Clausznitzer, D., Micali, G., Neumann, S., Sourjik, V. & Endres, R. G. Predicting chemical environments of bacteria from receptor signaling. *PLoS computational biology* **10**, e1003870, doi:10.1371/journal.pcbi.1003870 (2014).

3 Sourjik, V. & Wingreen, N. S. Responding to chemical gradients: bacterial chemotaxis. *Current opinion in cell biology* **24**, 262-268, doi:10.1016/j.ceb.2011.11.008 (2012).

4 Block, S. M., Segall, J. E. & Berg, H. C. Adaptation kinetics in bacterial chemotaxis. *Journal of bacteriology* **154**, 312-323 (1983).

5 Krembel, A., Colin, R. & Sourjik, V. Importance of Multiple Methylation Sites in Escherichia coli Chemotaxis. *PloS one* **10**, e0145582, doi:10.1371/journal.pone.0145582 (2015).

6 Walker, G. M., Monteiro-Riviere, N., Rouse, J. & O'Neill, A. T. A linear dilution microfluidic device for cytotoxicity assays. *Lab on a chip* **7**, 226-232, doi:10.1039/b608990a (2007).

7 Yuan, J., Fahrner, K. A., Turner, L. & Berg, H. C. Asymmetry in the clockwise and counterclockwise rotation of the bacterial flagellar motor. *Proceedings of the National Academy of Sciences of the United States of America* **107**, 12846-12849, doi:10.1073/pnas.1007333107 (2010).

8 Yang, Y. *et al.* Relation between chemotaxis and consumption of amino acids in bacteria. *Molecular microbiology* **96**, 1272-1282, doi:10.1111/mmi.13006 (2015).
